# Bioinformatics for precision medicine in oncology: principles and application to the SHIVA clinical trial

**DOI:** 10.3389/fgene.2014.00152

**Published:** 2014-05-30

**Authors:** Nicolas Servant, Julien Roméjon, Pierre Gestraud, Philippe La Rosa, Georges Lucotte, Séverine Lair, Virginie Bernard, Bruno Zeitouni, Fanny Coffin, Gérôme Jules-Clément, Florent Yvon, Alban Lermine, Patrick Poullet, Stéphane Liva, Stuart Pook, Tatiana Popova, Camille Barette, François Prud’homme, Jean-Gabriel Dick, Maud Kamal, Christophe Le Tourneau, Emmanuel Barillot, Philippe Hupé

**Affiliations:** ^1^Institut Curie, ParisFrance; ^2^INSERM U900, ParisFrance; ^3^Mines ParisTech, FontainebleauFrance; ^4^INSERM U932, ParisFrance; ^5^INSERM U830, ParisFrance; ^6^Institut Curie, Informatic Department, ParisFrance; ^7^Institut Curie, Sequencing Facility ICGex, ParisFrance; ^8^Institut Curie, Translational Research Department, ParisFrance; ^9^Department of Medical Oncology, Institut Curie, ParisFrance; ^10^CNRS UMR144, ParisFrance

**Keywords:** precision medicine, clinical trial, bioinformatics, sequencing, oncology, SHIVA

## Abstract

Precision medicine (PM) requires the delivery of individually adapted medical care based on the genetic characteristics of each patient and his/her tumor. The last decade witnessed the development of high-throughput technologies such as microarrays and next-generation sequencing which paved the way to PM in the field of oncology. While the cost of these technologies decreases, we are facing an exponential increase in the amount of data produced. Our ability to use this information in daily practice relies strongly on the availability of an efficient bioinformatics system that assists in the translation of knowledge from the bench towards molecular targeting and diagnosis. Clinical trials and routine diagnoses constitute different approaches, both requiring a strong bioinformatics environment capable of (i) warranting the integration and the traceability of data, (ii) ensuring the correct processing and analyses of genomic data, and (iii) applying well-defined and reproducible procedures for workflow management and decision-making. To address the issues, a seamless information system was developed at Institut Curie which facilitates the data integration and tracks in real-time the processing of individual samples. Moreover, computational pipelines were developed to identify reliably genomic alterations and mutations from the molecular profiles of each patient. After a rigorous quality control, a meaningful report is delivered to the clinicians and biologists for the therapeutic decision. The complete bioinformatics environment and the key points of its implementation are presented in the context of the SHIVA clinical trial, a multicentric randomized phase II trial comparing targeted therapy based on tumor molecular profiling versus conventional therapy in patients with refractory cancer. The numerous challenges faced in practice during the setting up and the conduct of this trial are discussed as an illustration of PM application.

## INTRODUCTION

### ERA OF PRECISION MEDICINE

Though physicians have always considered the individual characteristics of each of their patients, the term personalized medicine appeared recently to account for our new abilities to characterize each person biologically with genomic analysis, and to use this information to guide medical decision-making and deliver the best treatment to each patient. This concept is also referred to as genomic medicine, and other terms such as stratified medicine or targeted medicine are sometimes used interchangeably. A few years ago, the concept of P4 medicine was introduced with the idea of managing the patient’s health instead of the patient’s disease (Hood and Friend, 2011). As a matter of fact, the practice of medicine today is mainly reactive, i.e., the physician treats the patient’s disease and little is done to prevent the occurrence of the disease. The P4 medicine considers a model of healthcare that is predictive (considering the genetic background of the individual and his/her environment), preventive (adapting lifestyle, taking prophylactic drugs), personalized (tailoring the treatment to the individual’s unique features, such as the patient’s genetic background, the tumor’s genetic and epigenetic landscape, his/her life environment) and participatory (many options about healthcare which require in-depth exchanges between the individual and his/her physician). P4 medicine therefore extends the concept of personalized medicine.

The term precision medicine (PM) is also frequently encountered in the literature to denote similar ideas, and generally refers to delivering the right drug at the right time to the right patient, by targeting specifically the molecular events that are responsible for the disease. We will use in this article the terminology PM defined as a customization of healthcare that takes into account individual differences among patients from prevention, diagnosis, prognosis, choice of the treatment and follow-up. PM combines the knowledge of the patient’s characteristics with traditional medical records and environmental information to optimize health. PM does not only rely on genomic medicine but also integrates any other relevant information such as non-genomic biological data, clinical data, environmental parameters and the patient’s lifestyle.

### PM IN ONCOLOGY

In a special issue, the Journal of Clinical Oncology has focused on PM in oncology (Garraway et al., 2013) showing that this new era of medicine offers new perspectives to cure cancer. PM also raises numerous challenges including biobanking, bioinformatics and legal issues (Garraway et al., 2013; Meric-Bernstam et al., 2013; Overby and Tarczy-Hornoch, 2013; Suh et al., 2013). The intrinsic complexity of cancer and the variety of its forms (each tumor being genetically unique) designate this pathology as a prime target for PM approaches. Cancer is a disease caused by the accumulation of mutations occurring in critical genes (oncogenes and tumor-suppressor genes) and resulting in the alteration of key molecular pathways. Due to the genetic nature of cancer, the oncology research has largely benefited from the advances in high-throughput genomics technologies in order to decipher the molecular alterations involved in the tumorigenesis on one hand, and to help the clinician to tailor the therapy on the other (Tamborero et al., 2013). Molecular profiling based on genomics information from the tumoral DNA and constitutional DNA offers new insights into the prediction of the disease progression and the response to treatment for each individual patient. These approaches are based in particular on two dominant concepts: oncogene addiction and synthetic lethality. The first one, oncogene addiction, stipulates that some tumors rely on one particular oncogene for their survival and progression, and inhibiting this gene would therefore stop tumor growth; this is the magic bullet idea introduced by Paul Ehrlich in 1900. The second one, synthetic lethality, refers to the observation that the inactivation of a pair (or more) of genes might be lethal, whereas individual inactivation of any of these genes would not kill the cell. It offers an opportunity to selectively kill cancer cells, if they already present gene inactivation for one gene of the synthetic lethal pair, by targeting the second gene of the pair. A famous example is the synthetic lethality of *BRCA* and *PARP* genes, which is exploited by using *PARP* inhibitors for treating *BRCA* deficient breast cancer tumors. Both oncogene addiction and synthetic lethality are typical situations where targeted therapy should be the solution of choice.

The identification of genomic alterations used as biomarkers along with the emergence of molecularly targeted agents (MTAs) such as tyrosine-kinase inhibitors have promoted the development of PM in oncology. MTAs have proven their efficacy in some cancer subtypes and they provide new opportunities to treat the disease (see Dienstmann et al., 2013, for a review). The first MTA has been trastuzumab, which is a monoclonal antibody targeting the *ERBB2* receptor. This gene is amplified in 15–20% of patients with breast adenocarcinoma. Treating patients with locally advanced disease with trastuzumab for a year decreases by 50% the risk of recurrence (Piccart-Gebhart et al., 2005). Targeting the *BCR/ABL* fusion gene (i.e., the Philadelphia chromosome) with another MTA, imatinib, in patients with chronic myelogenous leukemia has dramatically improved their outcome (Druker et al., 2001). *BRAF(V600E)* mutation is frequently associated with melanoma, where it seems to play a critical role in the malignancy process and can be effectively treated using vemurafenib (Flaherty et al., 2010). *BRAF(V600E)* mutation has been also identified in multiple forms of advanced cancers such as colorectal or thyroid cancer (Cantwell-Dorris et al., 2011). It is generally accepted today that using MTA has great potential in the treatment of many types of cancer. Around 40 MTAs have been approved to date for the treatment of cancer and the development of new inhibitors is in progress. Developing new MTAs imply also to decipher new biomarkers among the large number of genomic alterations observed in tumors (mutations, amplifications, deletions, translocations, fusions and other structural variants). A large number of genomic alterations are passengers while very few are drivers. A subset of these drivers are actionable, i.e., have significant diagnosis, prognosis, or therapeutic implications in cancer, and a subset may also be druggable, i.e., targets for therapeutic development (Dancey et al., 2012). Classifying these genomic alterations into actionable and/or druggable is difficult and high-throughput screening techniques might help this classification. The possibility to search within each tumor the actionable/druggable alteration using high-throughput technologies opens the way to PM in the field of oncology.

### HIGH-THROUGHPUT SCREENING TECHNOLOGIES FOR PM

During the last two decades, the advent of high-throughput technologies has allowed the genome-wide characterization of molecular profiles in tumors. Among the different techniques, the gene-expression microarrays have been widely used so far in particular to build signatures for diagnostic and prognostic purposes. These gene signatures are now proposed as clinical tools for some types of breast cancer, for example Agendia’s 70-gene Agilent-based MammaPrint^®^, i.e., the Amsterdam Signature (van’t Veer et al., 2002; van de Vijver et al., 2002), Veridex’s 76-gene signature, i.e., the Rotterdam Signature (Wang et al., 2005; Foekens et al., 2006), Genomic Health’s 21-gene RT-PCR-based Oncotype DX^TM^ (Cobleigh et al., 2005; Hornberger et al., 2005) and a 41-gene expression set (Ahr et al., 2001; Molecular, Ahr et al., 2002). Ten years ago, next-generation sequencing (NGS) technology appeared. It has evolved so quickly that it is possible today to sequence a genome for a few thousand dollars within a few days. Of note, the sequencing of the first human genome costed around 3 billion dollars and took more than 10 years to be completed in 2003. The ability to simultaneously sequence millions of short nucleic acid fragments in parallel in a very short time and at very competitive costs (Sboner et al., 2011) makes NGS a major tool in oncology (Tran et al., 2012). NGS will very likely replace microarrays in a near future both for research and clinical applications. The current NGS techniques allow the profiling of the transcriptome (RNA-seq), the genome (DNA-seq, exome-seq), the epigenome (bisulfite-seq), the identification of DNA-protein interactions (ChIP-seq) and the reconstruction of chromosome architecture (Hi-C). While some sequencing platforms are very suitable for research purposes, the long duration of runs as well as the cost of these instruments are clearly incompatible with a real-time application for clinical use (e.g., the HiSeq sequencer from Illumina which tends to become the reference for very high-throughput sequencing, requires approximately 11 days per instrument and per run to generate data). In response to these concerns, benchtop sequencers were introduced such as the MiSeqDx^TM^ from Illumina or the Ion Torrent^TM^ PGM from Life Technologies. Benchtop sequencers allow the sequencing of a few megabases in a couple of hours with a very high depth of coverage. Their relatively low cost and rapid turnaround time make them very suitable for clinical applications. In November 2013, the MiSeqDx^TM^ was the first sequencer obtaining clearance from the Food and Drug Administration for clinical use as this platform demonstrated its precision and reproducibility across instruments, users, days and reagent lots (Collins and Hamburg, 2013). Benchtop sequencers make it possible to sequence rapidly fractions of the genome (target-seq) like the coding regions or a subset of known genes or mutation hotspots. The target-seq offers the possibility to screen several hundred mutation hotspots located in tumor-suppressor genes and oncogenes using dedicated cancer panel kits. Thus, the target-seq techniques offer new opportunities for diagnosis and many laboratories are shifting from Sanger sequencing to NGS platforms in order to meet challenges in terms of throughput and turnaround time. As an example recent advances have been made in the screening of the *BRCA1* and *BRCA2* genes and the detection of germline mutation related to an increased risk of developing breast cancer (Bosdet et al., 2013; Tarabeux et al., 2013).

### FRAMEWORK FOR PM IN ONCOLOGY

**FIGURE 1:**
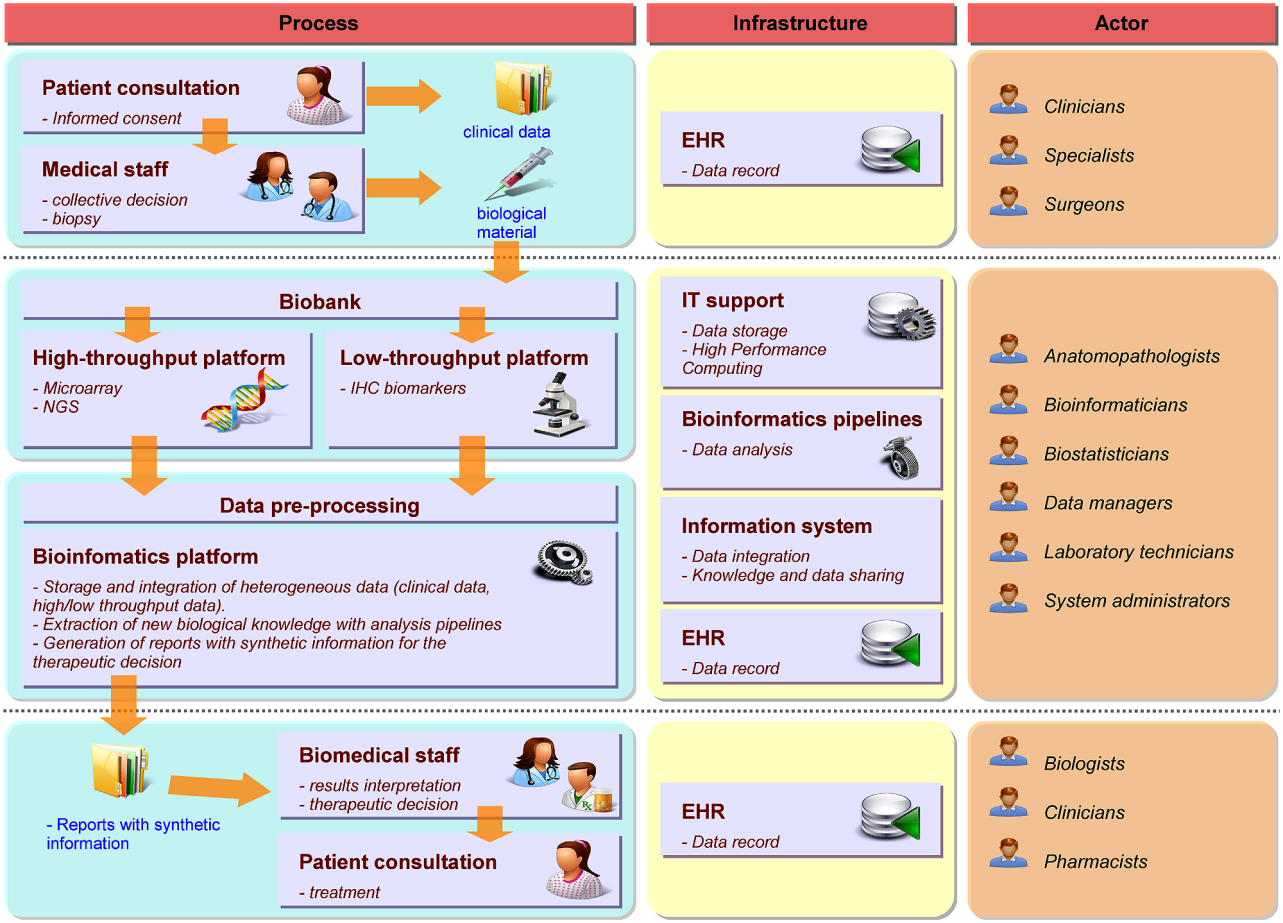
**Framework for PM in oncology.** The left part describes the workflow and processes required for the decision-making from patient consultation to the therapeutic decision. The middle part focuses on the informatics and bioinformatics architecture required to support the different steps of the workflow. The right part indicates the different experts involved in each process.

Precision medicine requires a strong interdisciplinary collaboration between several stakeholders covering a large continuum of expertise ranging from medical, clinical, biological, translational, technical, and biotechnological know-hows. **Figure 1** illustrates the different practitioners involved in the complex process, describes the data workflow starting from and coming back to the patient in order to tailor the therapy and shows the informatics and bioinformatics infrastructure supporting the workflow. To build the therapeutic decision, the most exhaustive data ranging from clinical to biological, environmental and family information (e.g., description of the tumor histology, list of previous treatments, family history, etc.) needs to be collected along a complex healthcare pathway. As the disease evolves, new experiments such as high-throughput screens (with microarray or NGS technologies for example) or biomarkers detection by immunohistochemistry (IHC) have to be performed to measure relevant biological information required to choose the best therapy. During the process, physicians (including different specialists such as surgeons, pathologists, radiation and medical oncologists, etc.), biologists, pharmacists, bioinformaticians, computational biologists, biostatisticians, informaticians, biobank managers, biotechnological platform managers, clinical research associates, and the technical staff will offer their expertise for the benefit of the patient. Different actors and cultures and a variety of miscellaneous constraints, including meeting the deadlines for results delivery, render the application of PM in daily clinical practice extremely challenging. Organizational aspects are therefore essential for the success of PM (Veltman et al., 2013). Downing et al. (2009) mentioned the importance of Electronic Health Record (EHR) and Clinical Decision Support (CDS) for care delivery due to the acceleration of knowledge discovery and its impact on the increasing number of possible clinical decisions. Development in CDS is required to handle the large heterogeneity of data and their complexity. The authors also pinpoint the fact that PM strongly depends on our ability to collect, disseminate and process complex information. Indeed, every stakeholder produces information during the healthcare pathway at different time points and in different places. The overall information needs to be gathered, integrated and summarized in a digest report to facilitate the therapeutic decision-making.

### NEED FOR BIOINFORMATICS SOLUTIONS TO SUPPORT PM

The availability of high-throughput technologies dedicated to clinical applications makes it very attractive for cancer centers to use these new tools on a daily basis. However, establishing such a clinical facility is not a trivial task due to the aforementioned complexity of PM framework along with the overwhelming amount of data. Indeed, the field of oncology has entered the so-called big data era as the particle physics did several years ago. From the big data 4 V’s perspective, data integration issue (i.e., merging heterogeneous data in a seamless information system) in oncology can be formulated as follows: a large *Volume* of patients’ data is disseminated across a large *Variety* of databases which increase in size at a huge *Velocity*. In order to extract most of the hidden *Value* from these data we must face challenges at: (i) the technical level to develop a powerful computational architecture (software / hardware), (ii) the organizational and management levels to define the procedures to collect data with highest confidence, quality and traceability, and (iii) the scientific level to create sophisticated mathematical models to predict the evolution of the disease and risks to the patient. Obviously, an efficient informatics and bioinformatics architecture is definitely needed to support PM in order to record, manage and analyze all the information collected. The architecture must also permit the query and the easy retrieval of any data that might be useful for therapeutic decision in real-time thus allowing clinicians to propose the tailored therapy to the patient in the shortest delay. Therefore, bioinformatics is among the most important bottlenecks towards the routine application of PM and several challenges need to be faced to make it a reality (Fernald et al., 2011). First, the development of a seamless information system allowing data integration, data traceability, and knowledge sharing across the different stakeholders is mandatory. Second, bioinformatics pipelines need to be developed in order to provide relevant biological information from the high-throughput molecular profiles of the patient. Third, the architecture must warrant the reproducibility of the results.

If many recent publications point out the key role of the bioinformatics for PM today (see Simon and Roychowdhury, 2013 for a review), clinical trials usually do not detail the complete bioinformatics environment used in practice to assess the quality and the traceability of the generated data. Different software platforms such as transMART (Athey et al., 2013), G-DOC (Madhavan et al., 2011) or the cBio Cancer Genomics Portal (Cerami et al., 2012) have been recently developed to promote the data sharing and analysis of genomics data in translational research. Canuel et al. (2014) reviewed the different solutions available and compared their functionalities. One of the most interesting features of these platforms relies on their analytical functionalities. They provide ready-to-use tools through user-friendly interface offering interesting functionalities for data queries and user analysis. However, these different solutions do not address essential aspects which are offered by our system: first, often they handle a specific type of data; second they do not cover management and traceability of the data in real-time as long as they are generated by the different stakeholders; third they do not provide clinicians with a meaningful digest of the analyses, which they need to take clinical decisions.

In the next section, we will focus on the bioinformatics solutions implemented in order to tackle these challenges in the Institut Curie Bioinformatics platform in the context of the SHIVA clinical trial (Le Tourneau et al., 2012) initiated in October 2012 at Institut Curie (Paris, France). This trial provides a concrete and practical application of a PM project. First, we will describe the design of the SHIVA clinical trial. Second, the seamless information system we have implemented to manage data along with the bioinformatics pipelines used to deliver the results for the therapeutic decision will be presented. Finally, the ongoing challenges will be listed.

## DESIGN OF THE SHIVA CLINICAL TRIAL

**FIGURE 2:**
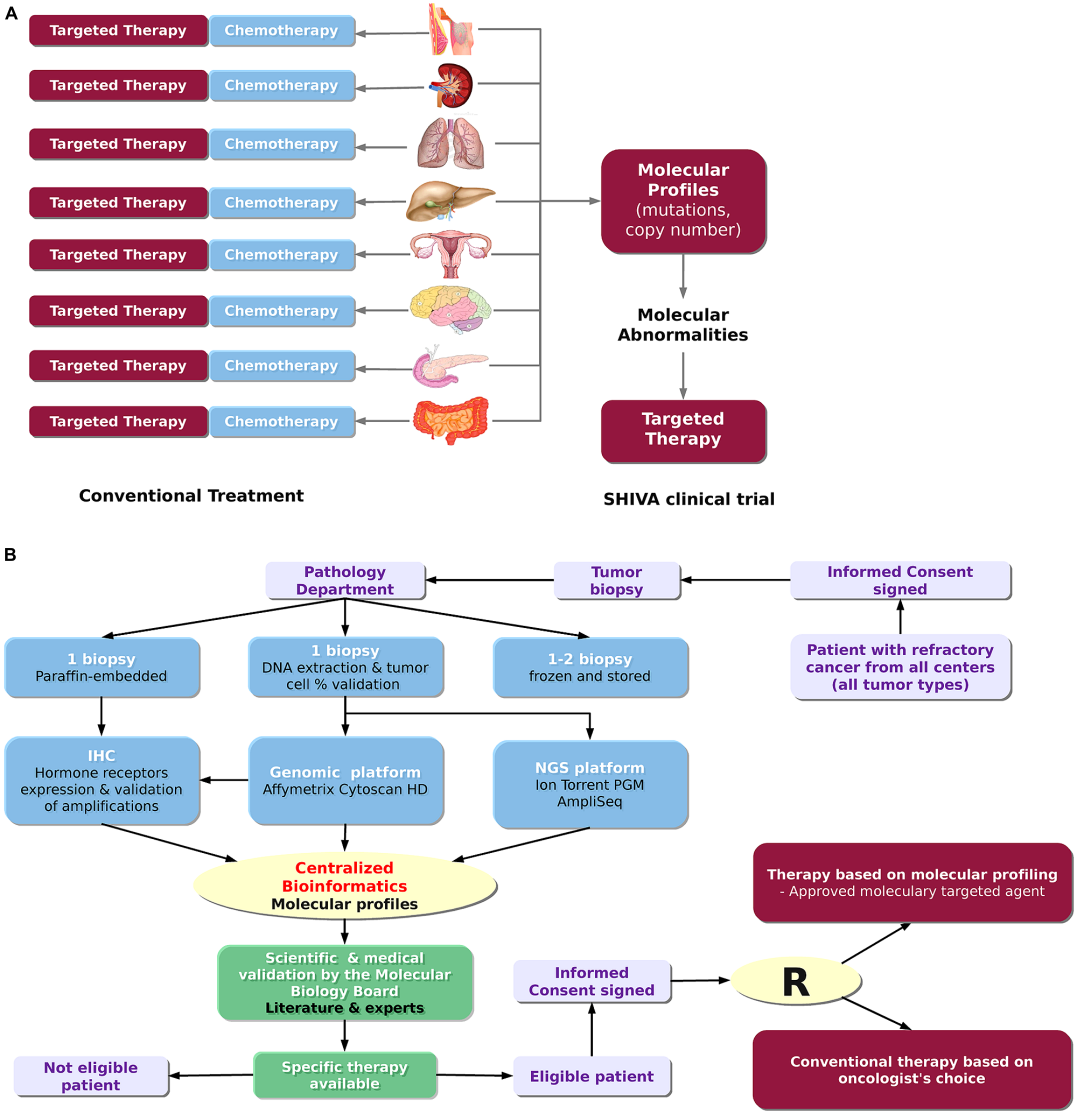
**The SHIVA clinical trial. (A)** Rationale of the trial. The SHIVA trial aims at determining whether the prescription of molecularly targeted therapies based on tumor molecular abnormalities, independently of primary tumor location and histology, would improve the outcome of cancer patients. **(B)** Design of trial (adapted from Le Tourneau et al., 2012). The SHIVA clinical trial involves many different actors from the patient inclusion to the therapeutic decision. The whole process requires less than four weeks including 4 days for the bioinformatics treatment.

SHIVA is a randomized proof-of-concept phase II trial comparing molecularly targeted therapy based on tumor molecular profiling versus conventional therapy in patients with refractory cancer^1^ (**Figure 2A**; Le Tourneau et al., 2012). Randomized trials in oncology are usually performed in a homogeneous population of patients with a specific tumor type and in a specific setting. In contrast, the goal of the SHIVA clinical trial is to bring the proof-of-concept that the prescription of molecularly targeted therapies based on tumor molecular abnormalities, independently of primary tumor location and histology, would improve the outcome of cancer patients. Therefore, all tumor types are allowed in the trial (n.b. no more than 20% of patients with the same primary tumor location will be randomized). Both DNA copy number alterations and mutations in a subset of 76 genes are considered for the decision-making. These genes cover in particular three main signaling pathways: (1) the hormone receptors pathway, (2) the *PI3K/AKT/mTOR* pathway, and (3) the *MAP* kinase pathway. They include predictive biomarkers of efficacy of the MTAs as well as known biomarkers of resistance (e.g., *KRAS*). These predictive biomarkers had either been validated in the clinic (e.g., *ERBB2* amplification for anti-ERBB2 therapy, Piccart-Gebhart et al., 2005) or been supported by strong preclinical study (e.g., *PI3KCA* mutations for mTOR inhibitors, see Carew et al., 2011, for a review). Of note, not all of the 76 genes are of interest for the SHIVA trial but the whole panel includes mutations that might be of interest for non-randomized patients who may be eligible for clinical trials based on not yet approved MTAs. For each patient, a biopsy from the metastasis is performed and the molecular profiles are assessed using both the Cytoscan HD technology (Affymetrix) for the detection of DNA copy number alterations and loss of heterozygosity (LOH), and the Ion Torrent^TM^ PGM sequencing technology (Life Technology) for the detection of somatic mutations. IHC is used for the assessment of hormone receptor status, including estrogen, progesterone and androgen receptors, as well as for the validation of focal gene amplifications detected with Cytoscan HD in the following genes: *ALK*, *BRAF, EGFR*, *ERBB2*, *KIT*, *MET*, *PDGFRA, PDGFRB *and* PTEN*. Only samples which contain more than 30% of tumor cells are processed to control at best sample heterogeneity. Patients from seven hospitals in France can be included in the study. The establishment of the molecular profiles follows the process and the timelines described in **Figure 2B**. All the bioinformatics steps including data management and integration, molecular profile analyses and data coherence checking, are centralized at the Institut Curie bioinformatics platform. This centralization permits the analysis of the molecular data from the different hospitals using the same parameters therefore ensuring the reproducibility of results. The whole process was set up in real-time in order to have less than four weeks elapsed between the biopsy and the randomization, including 4 days for the bioinformatics treatment (**Figure 2B**). Thus, this trial represents a concrete application of PM. It highlights the real challenges and difficulties about the feasibility of such project in real-time. A committee of expert named the Molecular Biology Board (MBB) has been appointed. It consists of biologists, bioinformaticians and medical oncologists of each hospital. The MBB meets each week to decide what the best therapy is for each patient. Based on its scientific expertise and a literature review, the MBB has defined a set of rules taking into account the relevant molecular abnormalities identified in the tumor to decide which MTAs to choose (among a list of 11 drugs) to treat the patient. MTAs allowed in trial are only drugs that are approved for clinical use in France. In the next section, we will describe the bioinformatics solutions we have developed at Institut Curie to manage the data workflow for the SHIVA clinical trial.

## BIOINFORMATICS ENVIRONMENT FOR THE SHIVA CLINICAL TRIAL

### SEAMLESS INFORMATION SYSTEM

**FIGURE 3:**
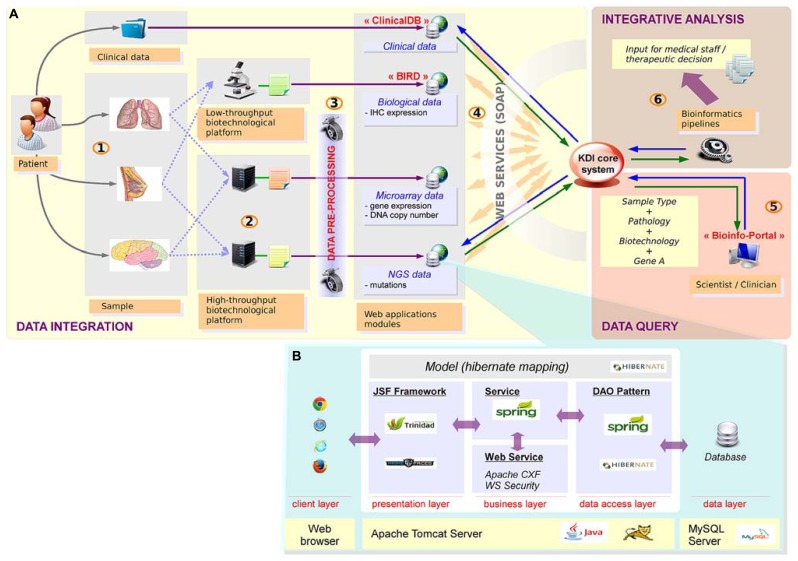
**Knowledge and data integration system. (A)** Integration of heterogeneous data requires a seamless information system with high scalability, plasticity, reliability and interoperability. (1) The samples are first collected for a given patient. (2) The samples are then processed by the technological platforms (NGS, microarrays, immunochemistry, etc.) (3) Raw data are analyzed using dedicated bioinformatics pipelines. Results are stored in dedicated applications. (4) Sample data and the corresponding patient are referenced in the KDI core system. (5) Advanced research functionality enable multiple data queries. (6) Specific bioinformatics pipelines generate new integrative knowledge from heterogeneous sources of data. **(B)** Technical view of web applications: web applications are based on n-tiers architecture, developed with J2EE technologies.

Precision medicine relies on a tight connection between many different stakeholders. As the choice of the therapy is based on a combination of different information levels including clinical data, high-throughput profiles (somatic mutations and DNA copy number alterations) and IHC data, all this information related to a given patient needs to be gathered in a seamless information system. Data integration is definitely required and bioinformatics plays a central role in setting up this infrastructure. To tackle this challenge, we have developed a seamless information system named KDI (Knowledge and Data Integration) described in **Figure 3**. The KDI system ensures information sharing, cross-software interoperability, automatic data extraction, and secure data transfer. In the context of the SHIVA clinical trial, high-throughput and IHC data are sent by the different biotechnological platforms to the bioinformatics platform using standardized procedures for transfer and synchronization. Data are then integrated into the KDI system within ad-hoc repositories and databases. Metadata describing the data are stored in the KDI core database such as the patient identifier, the type of data (e.g., mutation screening, clinical data, DNA copy number profile) and the technology used (e.g., Affymetrix microarray, Ion Torrent^TM^ PGM sequencing). Each type of data is then processed by dedicated bioinformatics pipelines in order to extract the relevant biological information such as the list of mutations and the list of amplifications/deletions. Therefore, the KDI core database acts as a hub allowing referencing all data through the use of web services. The KDI core database knows exhaustively which data is available for a given patient and where the raw and processed data are physically stored. It thus offers the possibility for clinicians to make queries through a web application and to extract the list of available information for a given patient. In addition, the system is also used to manage and perform automatic integrative analysis required for the therapeutic decision.

From a technical point of view, the KDI system consists of different modules dedicated to the storage, processing, analysis and visualization of each type of data (clinical, biological, microarray, NGS, etc.). High modularity associated with an efficient interoperability makes our system able to retrieve any relevant information. To facilitate the developments of these modules, we have retained a classical n-tiers architecture implemented with the JAVA/J2EE language. The core of each module of the KDI system can be presented as the association of different layers (**Figure 3B**).

#### Data layer

Data are stored in a relational database using the Entity-Attribute-Value (EAV) pattern. This conceptual modeling provides a data model plasticity required to handle the heterogeneity and the scalability of the variables of interest. Therefore, with EAV modeling, same concepts managed by different projects (with specific requirements by project) can be stored in a unique database without any modification of the data model. MySQL has been chosen as database provider for all web applications of the system. Complementary solutions such as NoSQL databases are currently evaluated for particular requirements (ontologies storage, specific queries, etc.).

#### Data access layer

Data access is supported by the DAO (Data Access Object) pattern. By using HibernateDaoSupport superclass provided by Spring Framework, we promote the standardization of database access for all standard queries (findAll, findById, save, delete). Moreover, Hibernate mapping through JPA annotations associated with use of Hibernate Criteria provides a homogeneous frame for this critical layer. Database sessions and transactional aspects are also delegated to Spring Framework.

#### Business layer

Business core of our web applications has two main objectives: (i) provide structured data for presentation layer, and (ii) make data available for remote and secured access by other applications and technical users. Standard services are developed using core functionalities of Spring framework (Aspect-Oriented Programming - AOP, Inversion of Control - IoC, JavaBeans Factory). Web services are published (server side) and invoked (client side) through Apache CXF framework. To respect Web Services Security (WS-Security) standards, we use the Apache WSS4J project provided by CXF (with interceptors chain process) to set up a username token authentication on each web application in the system.

#### Front-end layer

Presentation layer is based on JSF (Java Server Faces) which is a component oriented framework for building user interfaces for web applications. To enrich the basic component set provided by JSF, we use additional component libraries such as Apache Trinidad and Primefaces. By this systematic approach for each user interface, we aim to build a visual identity, ergonomic, easily usable, for the whole information system. All data available within KDI can be browsed and retrieved from a user-friendly bioinformatics web portal.

#### Client layer

This layer represents the web browser through which end-users access KDI system.

### DNA COPY NUMBER ANALYSIS PIPELINE

**FIGURE 4:**
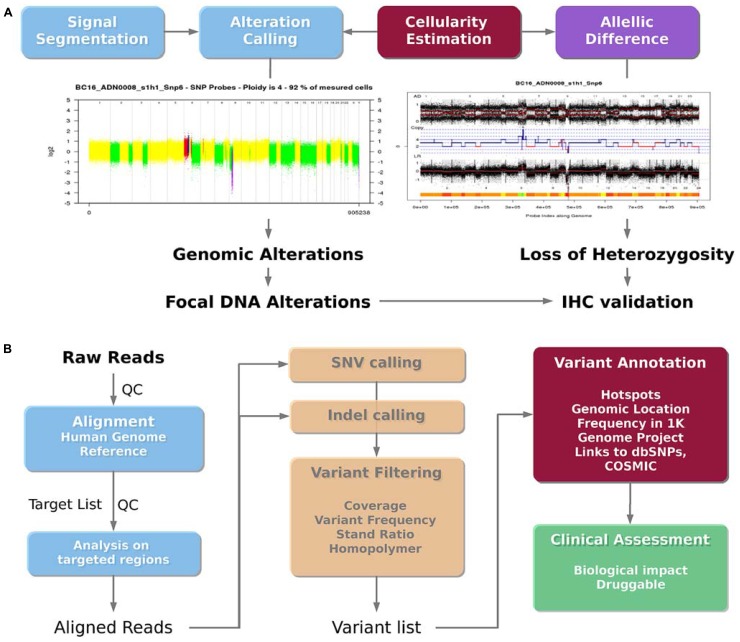
**Bioinformatics analysis pipelines. (A)** DNA copy number analysis pipeline. The DNA copy number signal is segmented and called to detect genomic alterations (deletion, loss, normal, gain and amplification) and loss of heterozygosity taking into account the sample cellularity. Focal gains or amplifications are then identified as potential druggable regions. **(B)** Mutation analysis pipeline.****The sequenced reads are aligned on the human reference genome, and centered on the targeted genomic regions. Single nucleotide variations (SNVs) and insertion/deletion (indels) are then called. The filtered variations can then be annotated using additional databases in order to lead to a final list of potential druggable variants.

The use of the Affymetrix CytoscanHD microarray allows both the detection of DNA copy number alterations and the loss of heterozygosity events. The analysis workflow is presented in **Figure 4A**. Raw data are normalized with the Affymetrix Power Tools software package^2^. Then, the log R ratio is segmented in order to detect breakpoints and assign copy number status using Colibri (Rigaill, 2010) and GLAD (Hupé et al., 2004) software. A similar process is applied on the allele difference profile using the GAP software (Popova et al., 2009). Both profiles (DNA copy number and LOH) allow the estimation of absolute copy number for each probe taking into account the sample cellularity and tumor ploidy estimated by the GAP algorithm. Each gene status (normal, gained, amplified, lost, deleted, loss of heterozygosity) can then be assessed. Copy number alterations are defined as follows: deletion = 0 copy, loss = 1 copy, normal = 2 copies, gain = 3, 4 or 5 copies and amplification ≥ 6 copies for diploid tumor, and deletion = 0 copy, loss = 1 or 2 copies, normal = 3 or 4 copies, gain = 5 or 6 copies and amplification ≥ 7 copies for tetraploid tumors. Additional steps in the analysis are performed to distinguish between large scale events such as chromosome arm gain and focal events targeting single oncogene or tumor-suppressor gene. Focal gains and amplifications are defined as genomic alterations with a size less than 10 Mb, and a copy number greater than the surrounding regions. In order to check whether a focal gain or an amplification of a size between 1 and 10 Mb induce a protein overexpression, a validation using IHC is performed. A report with the list of genes to be validated by IHC is automatically sent to the pathologists.

### MUTATION ANALYSIS PIPELINE

The bioinformatics pipeline presented in the **Figure 4B** was applied to detect somatic mutations from the Ion Torrent^TM^ PGM sequencer using the Ampliseq^TM^ cancer panel. Ion Torrent^TM^ PGM raw reads are aligned on the reference human genome hg19 using the TMAP aligner (v0.3.7 Life Technologies). The best mapping score for each read is used to detect misalignment. The standalone package of the Torrent Variant Caller (v2.2 Life Technologies) is then used to call variants (SNVs and indels) from the mapped reads. In the context of clinical trial, variants have to be filtered to promote a high specificity, in order to avoid any false positive mutations. Thus, detected variants are filtered according to their frequency (≥4% for SNVs and 5% for indels), strand ratio (≥0.2), and reads coverage (≥30X for SNVs and 100X for indels). In addition, SNVs and mainly insertions and deletions detected in the context of a repeated region or a homopolymer are double checked. Homopolymer and repeated regions are prone to contain recurrent false positive, because of the limitation of the Ion Torrent^TM^ PGM technology. In most cases, the variant is discarded if also detected in other patients from the same sequencing run. Otherwise, variations specific to a sample, even within a repeat context, are reported. To facilitate the interpretation of individual patient data for clinical trials, the filtered list of variants is then annotated using the ANNOVAR software (Wang et al., 2010). Common polymorphisms found on more than 1% of the ESP or 1K Genome project population as well as recurrent and neutral variants on hotspots are reported. These variants do not present any therapeutic interest but are good internal controls to ensure the quality of the sequencing data. The Catalog of Somatic Mutation in Cancer (COSMIC) is used to annotate the mutations detected at a hotspot position. Non targeted mutations in genes covered by the panel, being non polymorphic nonsense, missense or indels are also reported, even if it may be difficult to know whether the alteration is involved in deregulating a particular pathway and whether it is clinically relevant. However, more stringent frequency filtering are applied for these cases (frequency ≥10% for SNVs and 15% for indels) leading to a higher specificity. Then, relevant mutations and variations are visualized using the IGV browser (v.2.0.35, Thorvaldsdóttir et al., 2013). The visualization remains an important step to assess the overall quality of the variant call, by taking into account the reads coverage, the error rate in the flanking region, the mutation position across the targeted region and across reads supporting them.

### INTEGRATIVE ANALYSIS: THE REPORT FOR THE MOLECULAR BIOLOGY BOARD

**FIGURE 5:**
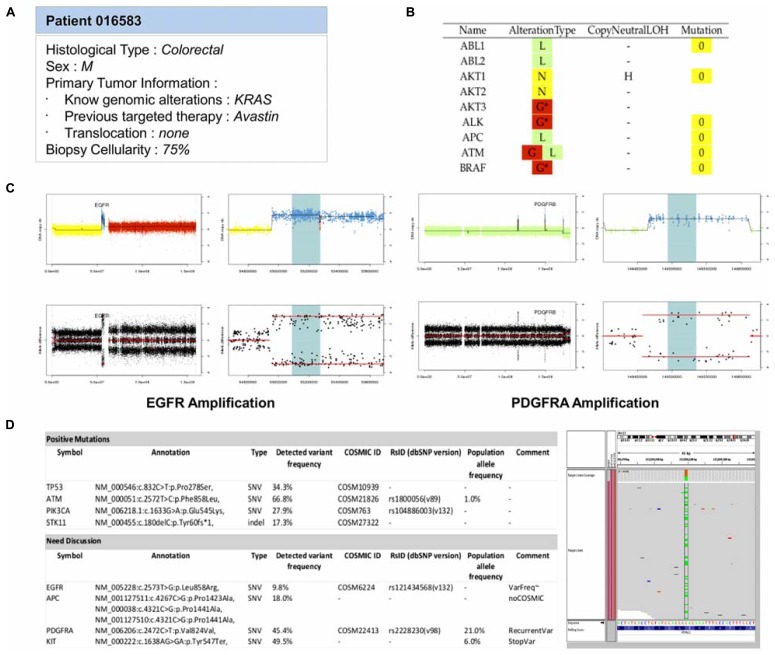
**Example of the main features reported in the MBB report. (A)** Patient information. All available clinical and biological informations about the patient are reported to guide the therapeutic decision. **(B)** Summary of molecular abnormalities. DNA copy number and mutations statuses for all genes are reported in a main table in order to provide a quick overview of the potential targets. The DNA copy number (D = Deletion, L = Loss, N = Normal, G = Gain, A = Amplification) and the mutations statuses are color-coded to ease the interpretation. **(C)** DNA copy number status. For each gene, detailed informations of their DNA gene copy number and loss of heterozygosity statuses are provided. The profiles are shown for two patients carrying an amplification of EGFR and PDGFRA. **(D)** Mutation status. Mutations passing all the filters are reported as positives mutations. Others cases can be discussed. In the following example, one variant does not have any COSMIC ID and has a frequency lower than 10%. Another is annotated as a recurrent variant, or outside an hotspot region. An IGV screenshot of the genomic region can help in validating the variant.

The last step of the bioinformatics workflow is the production a technical report for the MBB. This task is crucial and must be complete and precise on one hand, and summarized on the other to allow a quick decision of the board. To answer this need, a report is generated for each patient. This report first presents the clinical information of the patient and the overall molecular profiles per gene, with the DNA copy number alterations, LOH status, and number of mutations (**Figures 5A, B**). This first section provides the MBB with a rapid overview of all detected alterations. If needed, the MBB can also have access to more detailed results, with graphical views of the copy number profiles for each gene, as well as the list of mutations with detailed annotation as previously described (**Figures 5C, D**). This name-blinded technical report is sent to the members of the MBB for scientific validation and prioritization of the identified molecular abnormalities.

### SUMMARY OF THE DATA INTEGRATION WORKFLOW FOR THE SHIVA CLINICAL TRIAL WITHIN KDI

**FIGURE 6:**
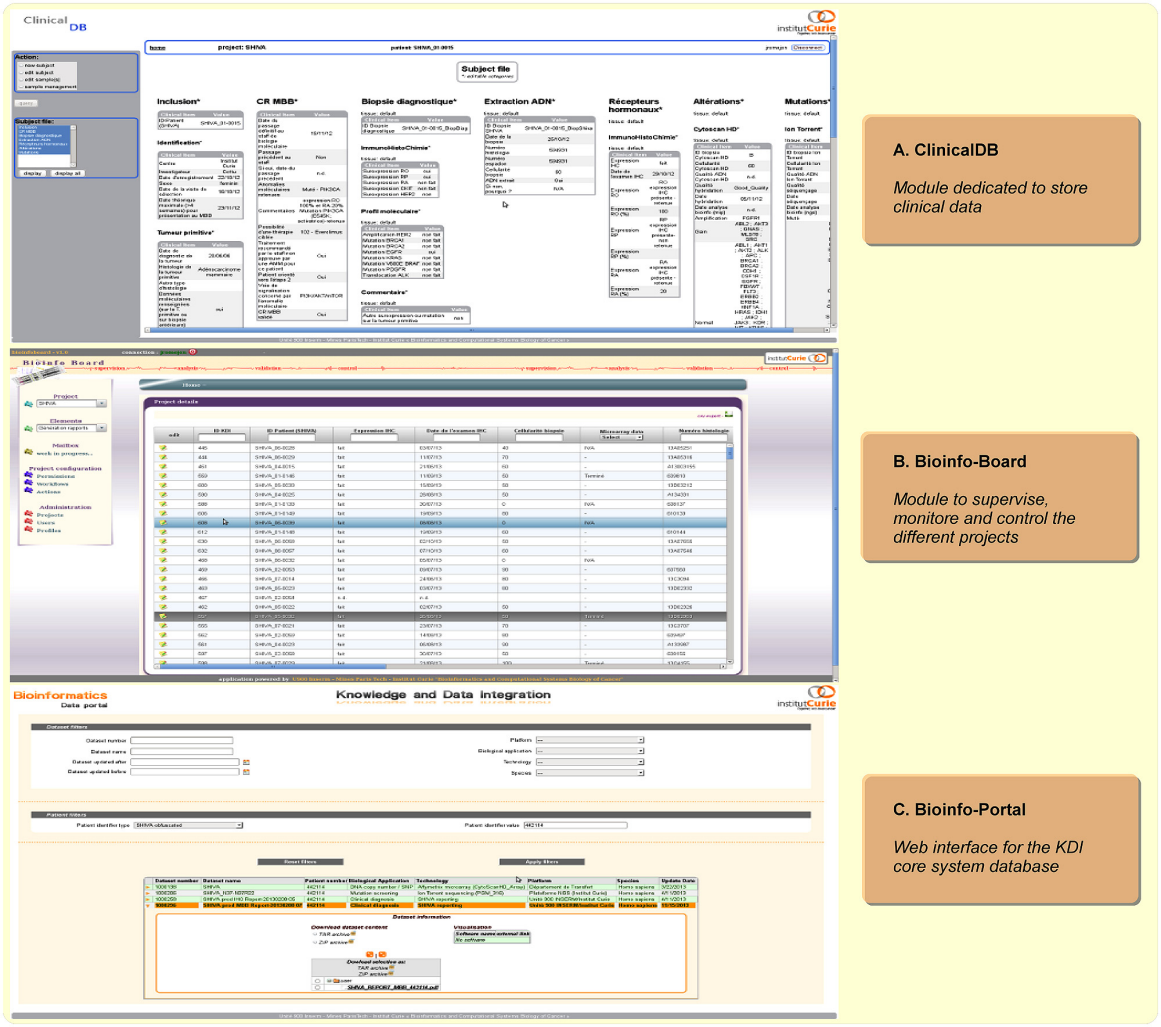
**Bioinformatics web applications screenshots. (A)** *Clinical DB* application is dedicated to clinical data storage. **(B)** *Bioinfo-Board* application aims to manage, and monitor each patient data workflow in real-time. **(C)** *Bioinfo-Portal* application is dedicated to the KDI core system database. It allows the access to all exported data and information for a given patient/project, such as data or clinical report in the context of the SHIVA clinical trial.

In the context of the SHIVA trial, the clinical data needed for the MBB are first imported in a dedicated module of the KDI system, named *ClinicalDB* (Clinical Database, **Figure 3A**). This step is performed weekly and updates the system by creating the patients recently included in the trial into the KDI core database. At the same time, an anonymous identifier is generated by the system for each new patient. Conversion between the different patient identifiers is guaranteed by the KDI core database and is accessible through the *Bioinfo-Portal* web application. Once available, the raw data generated by the biotechnological platforms are transferred to the bioinformatics platform for analysis (using rsync system). Bioinformatics pipelines (mutation and DNA copy number pipelines) process each molecular profile and are responsible for raw data storage and traceability within the KDI core database. The summarized results are structured in the *BIRD* (Biological Results Database) application. The last step of the data integration workflow is the generation of the bioinformatics reports. Two reports are required in the context of the SHIVA clinical trial at two different time points. A first report is generated by the system after the processing of the DNA copy number profile in order to request an IHC validation if needed. A second report is generated by the system and sent to the MBB for the final therapeutic decision. All reports, data and analysis results for each patient are gathered within the KDI modules (KDI core database, *ClinicalDB*, *BIRD*). All the information are available under controlled access for any member of the project through the KDI *Bioinfo-Portal*. In order to supervise the patients’ process at each step of the whole bioinformatics workflow, an additional module of the system named the *Bioinfo-Board* application (**Figure 6**) has been developed. This web application aims to controlling, monitoring and checking the evolution and status of each SHIVA patient in real-time.

### QUALITY MANAGEMENT

Offering a high quality service is most required in the context of a clinical application. The availability of all KDI’s component and the reproducibility of the analyses is thus mandatory. To this aim, we have promoted a set of good practices for the software building process. First, the software development phase follows a strict frame with positive technical constraints, and a common methodology known and shared by each data manager and software developer. The configuration management is delegated to a SVN repository where all the source codes of KDI system are regularly committed. The unit testing is strongly recommended for all programming languages involved in the system (X-Unit) and part of our continuous integration server based on Jenkins software. This system allows to check weekly that all the tests parameterized for all applications are successfully passed. This control ensures that the analysis pipelines provide the expected results, identical to a reference analysis which is considered as a gold standard. Second, we pay attention to the availability of KDI system. All web applications are monitored with Nagios software in order to be able to detect in real-time any disorder on the system and therefore take immediately all necessary actions (log analysis, server restart, update configuration, etc.) to restore initial and nominal state if needed.

In order to reach this high quality service expectations, three different informatics environments (meaning three different instances of all applications, three different web servers, three different database servers and three dedicated file systems) have been set up. An update on any environment is always linked with a SVN revision. The first environment is the development (D-env) which is the place of the version currently in development. Each developer, after doing unit testing on his local workspace is allowed to install a new version of his components on this environment. It results that the D-env can be temporarily unstable and this is assumed. The second environment is the validation (V-env) which must be stable at every time. Integration testing is performed on this environment to validate the candidate release of the KDI system. The V-env can be seen as a pre-production environment. Only the persons in charge of the final installation are allowed to update the V-env. The third environment is the production (P-env) which is the instance of KDI system really used by the end-users. The updates of the P-env have to be planned, secured, and widely announced to avoid any inconvenience. During delivery periods, the environments of validation and production must be identical. These three environments permit to secure our delivery process with a high reliability and traceability.

### FEASIBILITY OF THE SHIVA TRIAL

The presented bioinformatics environment is in use since October 2012 to manage and analyze the molecular profiles of the patients included in the SHIVA trial.

Results of the feasibility part of the project, focused on the first 100 patients were recently published (Le Tourneau et al., 2014). Among the first 100 patients, diagnostic confirmation and IHC analyses for hormone receptors were performed in 92% of the patients. Genomic analyses were performed for 65 patients (68%). DNA copy number analyses met quality criteria in all the 65 patients, while a technical problem occurred in 2 patients for mutations analyses. Overall, 58 out of the 95 patients (61%) had a complete molecular profile. All patient data were integrated in the KDI system. The median timeframe for the bioinformatics analysis (DNA copy number, mutation profiles and MBB report) was 5 days. Median timeframe from tumor biopsy/resection to MBB was 26 days [range: 14–42]. To date, eight French cancer centers are participating to the SHIVA trial, and more than 700 patients were included. All data provided by the different centers are centralized at Institut Curie using the KDI system, and analyzed in routine.

## ON-GOING CHALLENGES FOR PM

The solution we have developed to manage the SHIVA clinical trial provides a first step towards the routine application for PM. However, many challenges still need to be tackled and will require a lot of mutualization and harmonization efforts within the scientific community. The main on-going challenges are listed in what follows.

### COMPUTATIONAL ARCHITECTURE

Precision medicine does not only require an efficient informatics infrastructure at the software level but also at the hardware level. Indeed, as NGS is now widely used for tumor profiling, data processing relies on an efficient High Performance Computing (HPC) infrastructure for data storage, transfer, computation and access control. So far, mainly targeted sequencing on a limited panel of genes (e.g., using Ion Torrent^TM^ PGM with AmpliSeq^TM^) has been used and can be processed with relatively moderate computing resources. However, as sequencing cost keeps on decreasing, whole-exome or even whole-genome might be used soon thus requiring HPC infrastructure. According to Moore’s law, Kryder’s law and Butter’s law, costs are halved every 18, 12, and 9 months for processor, storage and data transfer, respectively (Stein, 2010) while 5 months was the rule for sequencing costs during the period 2007-2011 period (source^3^). Thus, the difference between biotechnological and informatics capacities grows exponentially. Entering the era of big data in cancer research implies a breakthrough at the informatics level. First, the scalability of the infrastrure (Input/Ouput performance and computing power) is required to allow the management and analysis of ever-growing data. Second, bioinformaticians must be trained to the use of low-level programming languages for parallel computing such as Message Passing Interface (MPI), Open MultiProcessing (OpenMP), or MapReduce (Dean and Ghemawat, 2008) and to the algorithm analysis. Developing these new skills will be essential in order to improve the efficiency of software used in downstream analysis to deliver results as quick as possible to meet deadline expected in the clinical practice. Third, the configuration of job scheduler (such as Torque/PBS, OGE, or Slurm) must ensure that resources could be available and allocated to analyze in priority the data needed for decision-making in clinic. This also implies a redundancy of the hardware components to ensure their availability. Resources and new know-how are definitely needed to handle NGS data and PM. Importantly, the question of which data and how long the data must be stored is an important issue. We can anticipate that at some point, the storage capacity will be lower than the amount of data generated meaning that data will have to be analyzed on-the-fly to extract the relevant information and reduce the volume.

### EXCHANGE STANDARDS AND ONTOLOGY

The large heterogeneity of the data that are collected along the healthcare pathway hampered their exchange and their comparison. Therefore, it is crucial to describe all the data that are generated with controlled vocabularies also called ontologies. Ontologies offer a formal representation of knowledge with definition of the relevant semantic attributes, their hierarchy and their relationship using a well-defined logic. Importantly, not only one single ontology can pretend to describe all the knowledge in a field but different ontologies (see^4^) are necessary to cover different entities of interest such as the gene (Gene Ontology), the disease (Disease Ontology) and the sequence (Sequence Ontology). Semantic Web standards promoted by World Wide Web Consortium (W3C) make it possible to link knowledge and data together so they can be queried and retrieved. To this aim, the Resource Description Framework (RDF) data format along with SPARQL query language provide the technical framework to describe, share, interact and query semantic data. While the technical solutions exist to support data exchange and linking, the definition of ontology, their choice and their use in practice for healthcare and biomedical data is still an issue. In order to tackle these challenges and to promote the use of standards and ontologies in the biomedical field, many European initiatives supported by the European Community (FP6 and FP7 programs) are involved in the definition and harmonization of standards:

*1. SemanticHEALTH* FP6^5^ focused on semantic interoperability issues of electronic health systems and infrastructures and provided a number of relevant definitions, standards, and application domains for semantic interoperability (Stroetman et al., 2009).

*2. SemanticHealthNet* FP7^6^ develops a scalable and sustainable pan-European organizational and governance process for the semantic interoperability of clinical and biomedical knowledge, to ensure that EHR systems are optimized for patient care, public health and clinical research across healthcare systems and institutions.

*3. p-medicine* FP7^7^ aims at developing new tools, data sharing and integration systems, IT infrastructure and Virtual Physiological Human (VPH) models to accelerate PM for the benefit of the patient.

Moreover, the European effort BioMedBridges supported by the European Strategy Forum on Research Infrastructures (ESFRI) aim to construct the data and service bridges needed to connect emerging biomedical sciences research infrastructures. Among the infrastructures concerned let us mention:

1. the European Infrastructure for translational medicine: EATRIS supports the development of biomedical discoveries for novel preventive, diagnostic or therapeutic products up to clinical proof of concept.

2. the Biobanking and Biomolecular Resources Research Infrastructure: BBMRI will form an interface between biological specimens and data and top-level biological and medical research.

3. the European Clinical Research Infrastructures Network: ECRIN supports multinational clinical research projects in Europe.

4. ELIXIR: (it aims to construct and operate a sustainable infrastructure for biological information in Europe to support life science research and its translation to medicine and the environment, the bio-industries and society.

### DEVELOPMENT OF SUSTAINABLE BIOINFORMATICS ANALYSIS PIPELINES

Maintaining an efficient bioinformatics workflow in the context of PM is today challenging because of the frequent updates of the computational solutions either installed on the sequencing machine or provided as standalone applications. These frequent updates are mainly due to the rapid evolution of the sequencing and microarray technologies but remain a major issue to ensure the operability of the bioinformatics pipelines and their reproducibility. As a consequence, any update requires that each bioinformatics pipeline is validated to warrant it provides a very high specificity and sensitivity. Indeed, any changes in the data format or in the analysis methods can have critical consequences on the downstream analysis and results. Moreover, many different methods are currently available to analyze NGS data but no consensus or standard computational tools exist so far. For instance, detecting germline or somatic mutations can be achieved using different bioinformatics algorithms, tools and filters. Choosing the most efficient algorithm is not an easy task and a feasibility phase is mandatory to define which algorithms and parameters to apply for a dedicated question.

### SAMPLE QUALITY CONTROL

The use of high-throughput technology in a clinical context also offers new challenges in the development of cutting edge statistical methods and algorithms dedicated to the field. As an example, the integration of heterogeneous molecular profiles provided by microarrays and sequencing assays could be used to define a patient genotype signature, to improve molecular profile accuracy and to ensure that the generated data come from the same biological samples and patient. The intersection of genotype variations available through the SNPs arrays technology could thus be intersected with the genotype information extracted from next-generation sequencing. However, this type of quality control requires the sequencing of a large DNA region to ensure that a sufficient number of polymorphism is covered. In the same way, the biopsy cellularity can also be estimated using both microarrays and sequencing assays (Larson and Fridley, 2013) in order to correlate the tumor purity from both profiles and detect intra-tumor heterogeneity.

### DEVELOPMENT OF DEDICATED COMPUTATIONAL AND MATHEMATICAL METHODS - TOWARDS SYSTEM MEDICINE

Clinical trials for PM rely so far on a very limited number of biomarkers used for the therapeutic decision (see Simon and Polley, 2013 for a review). Typically from one up to less than 50 biomarkers are used for PM in currently on-going clinical trials worldwide. Moreover, the decision is based on a univariate decision rule meaning that a possible interaction between biomarkers is not considered which certainly explains part of the limited efficacy of targeted therapies even in the presence of their targets. For example, Prahallad et al. (2012) showed that vemurafenib is highly effective in the treatment of melanoma in patients with *BRAF(V600E)* mutation while colon cancer patients harboring the same *BRAF(V600E)* mutation have a very limited response to this drug. They found that *BRAF* normally exerts a negative feedback regulation of *EGFR*. Therefore *BRAF* inhibition causes a rapid feedback activation of *EGFR*, which enhances cell proliferation. As melanoma cells express low levels of *EGFR* they are not subject to this feedback activation in contrast to colon cancer. Thus, they propose that these patients might benefit from combined therapy consisting of *BRAF* and *EGFR* inhibitors. This example highlights the fact that considering interactions between biomarkers and combining different therapies together can dramatically strengthen the efficiency of PM. Also it clearly shows that elucidating the reasons behind treatment escape and proposing backup therapeutic strategies would benefit greatly from the knowledge and modeling of the cell regulatory network rewiring. Therefore, computational systems biology approaches, based on mathematical models of the cell regulatory network rewiring, are definitely needed to deepen our understanding of the cancer cell and to improve current decision rules. Systems biology and systems medicine are two disciplines which open the road to PM. Machine learning techniques will also be very useful to develop prediction rules to predict outcome and response to treatment. We can imagine that online machine learning techniques could be used to refine and optimize decision rules as long as new data and knowledge are generated. The key defining characteristic of online learning is that soon after the prediction is made, the true label of the instance is discovered. This information can then be used to refine the prediction hypothesis used by the algorithm. In the case of cancer, every day, for several patients, information is collected: survival, response to therapy, molecular profiles, pathological complete response, etc. This information could be used to retrain the classifier on the available data. In addition to these data-driven approaches, knowledge-based approaches must be developed to capitalize on the large amount of knowledge that is present in the scientific and medical literature to build efficient decision rules. IBM has developed a supercomputer named Watson (the name of IBM’s founder) able to understand question in natural language and to extract relevant information from the literature. Watson supercomputer is currently used at the Memorial Sloan-Kettering (New-York, USA) to help for diagnosis in lung cancer.

### SEQUENCING THE GENOME AND BEYOND

Available NGS techniques expand from sequencing panels based on a couple of genes to whole-exome and whole-genome sequencing. Even if the whole-exome and whole-genome sequencing are currently used in cancer research, and can be seen as the future of the clinical investigation, their use in routine clinical practice is much more difficult, mainly because the average depth of coverage is much lower than for targeted genes sequencing complicating mutations detection. However, these applications offer new ways to explore DNA copy number and structural variations and can thus be used as an alternative to the current microarray technologies. In addition, the current sequencing capabilities also offer new opportunities to develop gene/transcript expression and epigenomics biomarkers in clinic. For instance, the detection of *BRCA1/BRCA2* isoforms and their quantification using RNA-seq approach would be an interesting complementary approach to mutations screening. In the same way, DNA methylation, histone modifications, small non-coding regulatory RNAs, or nucleosome remodeling regulate many biological processes involved in tumorigenesis. More recently, evidence that genetic and epigenetic mechanisms are related events in cancer has emerged. Alteration in epigenetic mechanisms can lead to somatic mutations, as well as somatic mutations in epigenetic regulators can lead to an altered epigenome (You and Jones, 2012; Timp and Feinberg, 2013). If drug discovery in cancer epigenetics had been held back due to concern about specificity and toxicity, it remains an active field of investigation (see Dawson and Kouzarides, 2012, for a review). The application of these new fields in clinic raises the question of combined therapies. Combination of targeted therapy with chemotherapy or with other targeted therapies is challenging because of increased toxicity. Solutions include the use of lower doses of drugs which might not be relevant if the biologically active dose is not reached and the use of drugs in a sequential manner although the relevance of this approach still needs to be demonstrated. For instance, it is likely that the combination of standard chemotherapy together with drugs against mutated proteins and epigenetics drugs offer synergetic benefits and increase therapeutic efficacy. Integrative analysis considering the multidimensional nature of the cancer (genome, proteome, epigenome, kinome, etc.) is therefore a major challenge to unravel the complexity of the disease and identify the most efficient treatments. To this aim, we will have to capitalize on large collection of public datasets such as data from The Cancer Genome Atlas (TCGA^8^, Kandoth et al., 2013) or International Cancer Genome Consortium (ICGC^9^) and also pathway databases for gene regulatory network, signaling pathway, metabolic pathway, Protein-Protein Interaction network and protein-compound network (e.g., DIP, HPRD, KEGG, Reactome to name only a few). The TCGA has initiated a pan-cancer analysis project (Cancer Genome Atlas Research Network et al., 2013) on the first 12 tumor types profiled by the consortium where the goal is to characterize molecular alterations and their functional impact across tumor type in order to promote the development of new therapies to fight cancer.

## CONCLUSION

We have developed a seamless information system named KDI that fully supports the essential bioinformatics requirements for PM. The system allows management and analysis of clinical information, classical biological data as well as high-throughput molecular profiles. It can deliver in real-time information to be used by the medical and biological staff for therapeutic decision-making. KDI makes it possible to share information and communicate reports and results across numerous stakeholders, representing a large continuum of expertise from medical, clinical, biological, translational, technical and biotechnological know-hows. The system relies on state-of-the-art informatic technologies allowing cross-software interoperability, automatic data extraction, quality control and secure data transfer. KDI has been successfully used in the framework of the SHIVA clinical trial for more than 18 months. KDI is also currently used for other clinical trials supported by European Union consortia covering cancer (RAIDs - Rational molecular Assessments and Innovative Drugs selection in cervival cancer) and non-cancer applications (MAARS - Microbes in Allergy and Autoimmunity Related to the Skin). This demonstrates the potentiality and flexibility of our system to support PM covering all its requirements ranging from data management, data traceability, data analysis, query, and visualization.

The evolution of sequencing technologies has expanded the frontiers of genomics in both biology and clinical environments. The sequencing field will continue to evolve rapidly, offering lower costs and increased speeds. On-going developments in the sequencing technology, such as an ultrafast sequencer like nanopore technology, will improve performance and miniaturization, thus offering new tools to improve prevention, diagnosis, prognosis, choice of the treatment and follow-up for patients in oncology. To promote PM in daily clinical routine, flexible bioinformatics systems like KDI are definitely required for enabling efficient sharing of information in real-time, and rapid data processing needed for therapeutic decisions. KDI also provides the infrastructure for developing and integrating into the clinical decision process new integrative analysis methods with sophisticated mathematical models, representing the multidimensional nature of cancer to propose new biomarkers and to develop new therapies to fight cancer.

## AUTHOR CONTRIBUTIONS

Nicolas Servant and Philippe Hupé coordinated the bioinformatics developments to support the SHIVA clinical trial. Philippe Hupé and Emmanuel Barillot coordinated the development for the seamless information system. Julien Roméjon, Philippe La Rosa, Georges Lucotte, Stéphane Liva, Alban Lermine, Virginie Bernard, Nicolas Servant managed the data and developed the bioinformatics pipelines for the SHIVA clinical trial. Virginie Bernard and Bruno Zeitouni developed the mutation pipeline for Ion Torrent^TM^ PGM. Pierre Gestraud, Philippe Hupé, Georges Lucotte, and Tatiana Popova developed the DNA copy number pipeline. Pierre Gestraud and Fanny Coffin developed the bioinformatics report for the molecular biology board. Philippe Hupé, Gérôme Jules-Clément, Florent Yvon, Patrick Poullet, Stéphane Liva, Alban Lermine, Stéphane Liva, Stuart Pook, Georges Lucotte, Philippe La Rosa, Camille Barette, and Julien Roméjon developed the seamless information system KDI. Camille Barette, François Prud’homme, Jean-Gabriel Dick managed the informatics infrastructure. Christophe Le Tourneau is heading the Phase I Program as well as the Head and Neck Clinic at the Institut Curie (Paris, France). Christophe Le Tourneau is the principal investigator of the SHIVA randomized personalized medicine trial. Maud Kamal is the scientific coordinator of the SHIVA trial. Nicolas Servant, Philippe Hupé, Emmanuel Barillot, Pierre Gestraud, Maud Kamal, Christophe Le Tourneau and Julien Roméjon wrote the article.

## Conflict of Interest Statement

The authors declare that the research was conducted in the absence of any commercial or financial relationships that could be construed as a potential conflict of interest.

## Footnotes

1 http://clinicaltrials.gov/show/NCT01771458

2 http://www.affymetrix.com

3 http://www.genome.gov/sequencingcosts/

4 http://bioportal.bioontology.org/

5 http://www.semantichealth.org

6 http://www.semantichealthnet.eu

7 http://p-medicine.eu

8 http://cancergenome.nih.gov/

9 http://icgc.org/
